# The Influence of the COVID-19 Pandemic on Orthodontic Treatments: A Survey Analysis

**DOI:** 10.3390/dj10020015

**Published:** 2022-01-20

**Authors:** Sabina Saccomanno, Stefano Saran, Elisabetta Guercio, Rodolfo Francesco Mastrapasqua, Alessio Pirino, Fabio Scoppa

**Affiliations:** 1 Department of Health, Life and Environmental Science, University of L’Aquila, Piazza Salvatore Tommasi, 67100 L’Aquila, Italy; 2 Dental School, Catholic University of the Sacred Heart, 00168 Rome, Italy; stefano.saran01@icatt.it; 3 Department of Orthodontics, Faculty of Dentistry, University Central of Venezuela, Caracas 1050, Venezuela; elisabetta.guercio@uniroma1.it; 4 ENT Department, Rivoli Hospital, ASL TO3, 10098 Rivoli, Italy; rfmastrapasqua@aslto3.it; 5 Department of Biomedical Sciences, University of Sassari, 07100 Sassari, Italy; axelpir@uniss.it; 6 Chinesis I.F.O.P. Osteopathy School, 00152 Rome, Italy; fabioscoppa@chinesis.org; 7 Master’s Degree Course in Posturology, Faculty of Medicine and Dental Surgery, University of Rome “Sapienza”, 00185 Rome, Italy

**Keywords:** orthodontic treatment, COVID-19 pandemic, facial mask, droplets, aerosol, distance learning, lockdown, psychological distress, virus SARS-CoV-2

## Abstract

Coronavirus disease 2019 represents the pandemic of the 21st century that has negatively affected the lives of the whole of humanity. For many months, the only weapons to fight against this infection were protective masks and social isolation. During recent months, fear of the virus has led people to avoid crowded environments and events, and to reduce medical checks, limiting them only to emergencies. Outpatient clinics, doctors’ offices, and all closed-in environments were required to limit the patients’ access. Nowadays, the presence of specific protocols around the world, and the extended vaccination campaign, have allowed a reduction of many restrictions. Unfortunately, the virus is still widespread, and dental practice and dental treatments suffer the consequences. Dental therapies in general, and in particular orthodontics, are not considered lifesaving. Due to this, orthodontists, in this historical time, must find solutions for motivating patients to start or continue therapies, while providing a safe way for them to do so. There are orthodontists who have developed, during this period, different ways to help them in treating and communicating with patients. **Aim:** The aim of this study is to assess the influence of the pandemic on the choice to start orthodontic treatment, oral health care, and the importance placed on the appearance of dental occlusions. **Materials and Methods:** This study is a survey analysis of 159 people, which was posted in Facebook groups of adult orthodontic patients. The timestamps and answers of responses were analyzed to avoid duplicated or interrupted questionnaires. **Conclusions:** This study found that the current health emergency has not reduced the demand for orthodontic care, while some patients’ behaviors are changing in relation to oral hygiene and the importance that they attribute to dental health. It seems that dentists’ availability plays a key role in this period of sanitary emergency.

## 1. Introduction

Coronavirus disease 2019, known as COVID-19, is the latest infectious disease which developed in the last two years. It mainly causes a severe acute respiratory syndrome, even if, in some cases, it affects other organs of the body. In the most complex cases, the patient can sustain significant long-term sequelae and death [1]. 

Diagnosis is clinical, confirmed by oropharyngeal or nasopharyngeal swab [2]. The speed, the spread, and the complications that a patient may face have created a health emergency that, for many months, led to periods of closures and lockdowns [3]. The consequences of these closures have led, in many sectors, to a collapse of the economy, a reduction of social life, and reduced care for people who, for fear of COVID-19 and hospitals, have reduced medical follow-ups [4]. The need for social distancing has led to a greater use of smart-working information technology, distance learning, and telemedicine [3,5,6]. 

All the restrictions of the current pandemic (use of masks, distance learning, social isolation, and lack of physical contact with relatives and friends) have also resulted in psychological problems in many people. Among adults, in particular, there has been an increase in issues such as depression, temporomandibular joint disorders [7,8], difficulty in recognizing others and oneself due to the use of masks, and self-neglect in people due to participating in smart-working and not being required to leave the house [9].

The pandemic started in 2020, and it is still ongoing, as of this writing. At the beginning of the pandemic, the first reaction of governments was to adopt drastic measures to flatten the curve of infection, to lower the impact of the virus on the health system [10].

This fact brought many changes to the health system during the first period of the pandemic. All non-urgent therapies and medical checks were postponed until the COVID-19 situation was predicted to be under control.

Dentists, according to “The Workers Who Face the Greatest Coronavirus Risk,” published by New Work Times, are the workers most exposed to the risk of being affected by COVID-19, much more so than nurses or general physicians [11,12]. 

General dentists, in almost all countries, were allowed to see patients only if it was medical urgent, while other therapies, that were deferrable, were delayed. One of these therapies was orthodontic treatment, which was left on standby. Moreover, in every case it was recommended to dentists that they take several personal protection measures, and avoid or minimize operations that produce droplets or aerosols [13]. 

Some orthodontist decided to have remote consults to check their patient’s situation, and help them if they had any kind of problem or doubts.

In the second half of 2020, the situation improved; the diffusion of facial masks and precautions taken to reduce the spread of COVID-19 helped, by allowing non-urgent therapies and medical checks to be scheduled and performed safely [12]. Furthermore, in 2021, when the vaccine started to be administered to people, the situation improved for vaccinated individuals, and the risk of developing a severe form of the disease decreased [3].

Dentists and orthodontists were able to see their patients again, with some precautions, and orthodontic therapies were able to be resumed [14,15]. 

However, there were patients who, because of fear of COVID-19 infection, continued to avoid hospitals and dental offices. This continued to happen even as it became clear that dental offices, due the precautions taken, are very safe, and the possibility of virus transmission is very low.

Orthodontic therapy is not lifesaving care, but it improves patients’ wellbeing, physically, socially, and psychologically. As a result of health risks and the economic crisis caused by COVID-19, many patients revised their priorities. In particular, it was recommended that vulnerable populations, and those who cannot be vaccinated for medical reasons, reduce social contacts and postpone non-urgent care. Fortunately, these populations are not often in need of early orthodontic therapy [16].

Orthodontic therapy is a kind of care which is very important for many aspects of health, and for some patients it cannot be postponed for a long time. These patients include growing children, who need regular checks to ensure the correct growth and development of their jaws and teeth. In a few cases, it is necessary to start therapy at a very young age to improve the pattern of development of these biological structures [10]. 

There is not the same sense of urgency for adults; however, there are other factors that increase their need to improve their smile and tooth appearance and alignment. It is evident that good alignment of the teeth can impact the quality of daily life from a psychological [17], social, and physical point of view [18]. It has been well documented how a good smile can improve personal relationships and self-confidence. Moreover, a good dental relationship can improve the function of the jaws, improving chewing and swallowing. Usually, adult patients ask for aesthetic devices; in particular, people who are in strict contact with the public sometimes decide to postpone or refuse orthodontic therapies because of the impact of oral devices on the aesthetic of their smiles [19]. 

Considering all that, this study aims to highlight how COVID-19 influences orthodontic therapy, access to the orthodontist’s office, orthodontists’ availability, the importance placed on this kind of treatment by people who are not experts in this field, and their capacity to afford its cost.

The purpose of this study is, as well, to see how the pandemic has affected the choice to begin an orthodontic treatment, requests for a specific type of device by patients, and how the clinician can, and should, manage the care process.

## 2. Material and Methods

An anonymized survey, available in two languages (Italian and English), was posted on select dedicated Facebook groups of adult orthodontic patients, where they share their experiences of orthodontic treatment. Certainly, the survey reached 175 patients all over the world, though only 159 responses were included in the study design. There were no reminders sent to patients, to let them feel free to answer. It was specified that the purpose of the questionnaire was to find ways for clinicians to improve their methods of curing patients. It was ascertained that each patient provided one answer by checking that the answers and timing were different. The patients were asked to complete the questionnaire without any possible compensation or benefit. The questionnaire was compiled for this study; due to the contingency of the pandemic waves, pre-testing was unviable.

We performed a study power analysis to estimate the minimum sample size needed, considering a power study of 0.8, alpha error of 0.05, and a difference of 20% in positive answers, with an optimal sample size of 181. We closed the study on February 2021, due to the end of the pandemic wave, and recruited a total of 159 responders between January and February 2021. 

All participants provided informed consent and accepted the privacy policy for the protection of personal data before completing the survey. No personal information that identifies the individuals was collected, and the data were analyzed in aggregate form only. All responses were collected on an anonymous basis, using the Google Form service. The resulting data file, used for data analysis, was free of any identifiers, including email and IP addresses, or other electronic identifiers. The study was conducted in accordance with the principles outlined in the Declaration of Helsinki.

All orthodontic patients aged 18 or older were included in the study. In the case of younger patients, elder relatives completed the survey. According to the structure of Italian syntax, the default gender used in questions was masculine, which includes both male and female orthodontists. 

The questions asked of the patients are the following:

○ How many times did you go to see your orthodontist in his office during the last year (the pandemic)?
○ During the pandemic did you go to your orthodontist in his office more or less than before?
○ Did you start your orthodontic treatment during the pandemic?
○ Did the pandemic influence your decision to go to the dental office to see an orthodontist?
○ Did the obligatory use of the facial mask to avoid COVID-19 infection influence your decision to have an orthodontic treatment?
○ Did the pandemic push you to ask for a specific kind of orthodontic device?
○ Did the pandemic influence the importance that you usually attribute to your smile appearance?
○ Did the pandemic influence the care that you usually give to your teeth health?
○ Did your oral hygiene change during the pandemic?
○ Did the uncertain economic situation caused by the pandemic, influence your decision to have an orthodontic treatment?
○ Did you decide to postpone or not to have an orthodontic treatment because of the current economic situation?
○ Did you feel a change in the orthodontist availability in satisfying your requests during the pandemic?

## 3. Statistical Analysis

All data points were analyzed using Statistical Package for the Social Sciences (SPSS 25.0, SPSS Inc., Chicago, IL, USA). Dichotomic data were analyzed using the Pearson chi-squared test, while three-choice questions were analyzed for correlation using the Spearman test. We performed a binary logistic regression for categorical variables confronted with binary outcomes. 

## 4. Results

The original sample reached by the survey was composed of 175 patients, but some were excluded because of duplicate or incomplete answers. The patients were from all over the world, which it is a factor that helps to provide a general view of the factors analyzed; around 20% of the people involved were from Italy. 

**Question correlations**

By analyzing the data, we found a significant association between “During the pandemic did you go to your orthodontist in his office more or less than before?” and “Did you start your orthodontic treatment during the pandemic?” (*p* < 0.01), as well as between “Did the pandemic influence your decision to go to the dental office to see an orthodontist?” (*p* < 0.01) and “Did you feel a change in the orthodontist availability in satisfying your requests during the pandemic?” (*p* < 0.01).

Comparing the questions: “Did the uncertain economic situation caused by the pandemic influence your decision to have an orthodontic treatment?” with “Did the pandemic push you to ask for a specific kind of orthodontic device?” showed a significance of *p* < 0.001.

People who stated, “It increased the importance of dental hygiene” in response to the question “Did the pandemic influence the care that you usually give to your teeth health?” showed a higher probability of starting a new orthodontic treatment during the pandemic (*p* < 0.05) (Table 1).

**Orthodontic checks**

A total of 43.5% of patients reported that the frequency of orthodontic checks did not change, while it increased for 21.4% of the sample. A total of 57.9% of the sample started an orthodontic treatment during the pandemic, while 76.8% of patients reported that the pandemic did not affect their decision to see an orthodontist. Only 11.3% reported that the pandemic discouraged them from going to the dental office, while 81.4% confirmed that even the mandatory use of facial masks did not influence their decision to undergo treatment (Table 2). Although 83.3% admitted that the importance of the aesthetics of their smile did not change for them, for 12.8% of participants it increased during the pandemic. A total of 92.5% of respondents affirmed that the pandemic did push them to ask for a specific orthodontic option, although, from the statistical analysis, it became evident that it was the economic situation that induced people to ask for specific kinds of devices (different types of braces, clear aligners, or movable appliances).

We did not find any correlations between the frequency of orthodontic visits and any other questions, including “Did the obligatory use of the facial mask to avoid COVID-19 infection influence your decision to have an orthodontic treatment?”, “Did the pandemic push you to ask for a specific kind of orthodontic device?”, “Did the uncertain economic situation caused by the pandemic, influence your decision to have an orthodontic treatment?”, “Did you decide to postpone or not to have an orthodontic treatment because of the current economic situation?”, “Did the pandemic influence the importance that you usually attribute to your smile appearance?”, “Did the pandemic influence the care that you usually give to your teeth health?”, and “Did your oral hygiene change during the pandemic?”.

For 83% of subjects, the pandemic did not influence the importance usually attributed to their smile appearance. A total of 75.5% of subjects reported that the pandemic did not influence the care that they usually dedicate to their oral health, although 18.9% reported that their usual dental care increased. Oral hygiene practices did not change during the pandemic for 64.8% of subjects, while they improved for 27.7%.

Smile importance and economic situation

The question “Did the pandemic influence the importance that you usually attribute to your smile appearance?” was significantly correlated (*p* < 0.01) to “Did the pandemic influence the care that you usually give to your teeth health?” and “Did the obligatory use of the facial mask to avoid COVID-19 infection influence your decision to have an orthodontic treatment?” (*p* < 0.01).

The uncertain economic situation caused by the pandemic did not influence the decision to undergo orthodontic treatment for 84.3% of responders, while it did for 15.1% of them. The availability of orthodontists to satisfy the patients’ requests during the pandemic did not change for 71.1% of responders, while it decreased for 22.6% of them.

A significant portion of the patients, specifically 20%, were Italian. The rest of the answers were from all around the world. We calculated the post-hoc power of the study for non-significant analyses, such as between “During the pandemic did you go to your orthodontist in his office more or less than before?” and “Did the uncertain economic situation caused by the pandemic, influence your decision to have an orthodontic treatment?”, estimating a prevalence of 10% and 20% of answers, and obtained a study power of 70% [1,2].

## 5. Discussion

Among other consequences of the COVID-19 pandemic, the fear of contracting the SARS-CoV-2 virus induced the majority people to avoid closed-in environments with many people, in particular hospitals and medical offices, where the risk of being infected was greater. However, based on the results we have seen during the pandemic, those who decided to accept the possible risks of being close to other people, including their orthodontists, were the ones who decided to receive orthodontic treatment. The same patients reported unchanged or increased availability of their orthodontist during the pandemic [20,21,22].

Conversely, those who felt that the availability of their orthodontist decreased went to dental clinics less often, and were the patients most weary and discouraged about beginning an orthodontic treatment, and even more in doubt about seeing a dentist (orthodontist). 

This aspect highlights how important it is for dentists and orthodontists to be accessible. The ability to communicate promptly is very important, in these historical times, to help patients feel safe, and the ability to show a greater availability to satisfy and solve the doubts of the patient is a key factor. To increase patients’ trust, it is very important to explain all the precautions taken by the dental office to avoid COVID-19 infections. 

From the results, it is evident that the current economic crisis, even in a limited sample, did not affect the decision to undergo orthodontic therapy, although there was a significant relationship between those patients who asked for a specific device and those who were influenced by their economic situation. That is a reasonable relationship, due to the different costs of various types of orthodontic devices, i.e., fixed braces, even if they impact appearance the most during treatment, are cheaper than lingual braces and aligners. 

It seems that the COVID-19 pandemic did not persuade people to postpone their therapy. Furthermore, the frequency of visits to the orthodontist did not change because of the economic situation, or because of the use of facial masks, even considering that facial masks may negatively affect people’s mood and reduce their social interactions. Moreover, the number of appointments necessary for orthodontic treatments did not change, possibly because of a temporary decrease in importance attributed to smile aesthetics and dental health. It can be reasonably deduced that facial masks can reduce attention to dental appearance and health [23].

From this survey, another important, though peculiar, aspect was revealed. It appears that those who were influenced by facial masks in their decision to treat their occlusion were the same group who started to be more careful to dental health and smile appearance. That can be explained by the fact that using masks led them be more aware of their teeth. 

It appears that, considering percentages, the majority of respondents were not influenced by using masks (81.4%) in deciding to undergo orthodontic treatments, while 18.9% reported that it was the use of masks that convinced them to take care of their teeth. This can be explained by the fact that facial masks can hide dental devices, which is, in many cases, the reason why some people refuse to be treated orthodontically. Considering that, the pandemic could be the perfect moment to receive orthodontic treatment for those people who pay close attention to their aesthetics. 

Another interesting aspect is that more than half of the sample started therapy during the pandemic, which was good news for orthodontists, who were worried about a possible significant decrease in patients, and for young patients who need to be treated early to solve skeletal malocclusions, which are best treated during growth to take full advantage of orthodontic principles.

Another positive aspect is the improvement of oral hygiene, which was related to 27.7% of the sample, and seems connected to requests for orthodontic therapy. This was an unexpected finding, considering the economic crisis, the psychological stress due to the pandemic, and the use of facial masks that hide the teeth. In fact, a small portion of the sample, 6.3%, reported a decrease in their attention to oral hygiene, which should push dentists to motivate their patients a bit more during the pandemic.

Further studies could be done to see which dental treatments were performed less frequently, and which medical check-ups were postponed.

It would also be useful to do another study when the pandemic ends, to verify if the choice to undergo orthodontic treatment is indeed, at least partly, related to the use of facial masks. The main limitation to this study is the short timeframe that was necessitated by the rapid socioeconomical changes of the pandemic. An important aspect, which is not highlighted in this article, it is the socioeconomic status of the patients, which, surely, affects their behavioural reaction to the crisis and life changes caused by the pandemic. The lack of complete socioeconomical status, and correlation with the pandemic status in responder countries, could have caused us to underestimate the effect of the pandemic and the different impact that the pandemic had on various countries, which affected patients’ life in heterogeneous ways. Finally, the formulation of the questionnaire itself is a limitation, as there are no validated items for this issue in the literature, and the contingency of the pandemic, characterized by short timespans between ebbs and flows of the infection and states responses, prevented us from conducting a preliminary validation.

## 6. Conclusions

This study suggests that the current health emergency is not reducing requests for orthodontic care, unlike in other medical areas. Considering at least this sample of responders, the economic crisis derived from this pandemic does not, in principle, affect orthodontists’ jobs, as it does not seem to affect the decision of most patients to undergo orthodontic treatment. Instead, the pandemic is changing some behaviors of patients in relation to oral hygiene, as well as the importance placed on dental health and appearance. These aspects are, in general, improving, even if they are not in a few cases, and it is necessary that dentists motivate their patients to take care of their teeth, even if they are almost always covered by facial masks.

Furthermore, the rigid anti-infection protocols of dentists, in relation to the great risk of working in the oral cavity and with saliva, certainly, together with the need to wear a protective mask (which hid the use of the device), encouraged patients to undergo orthodontic treatment.

Eventually, it seems that the availability of doctors plays a key role in this pandemic in promoting orthodontic therapy. Dentists play an important role, as he or she can affect their patients’ behavior by encouraging them to stay compliant. In these times, it seems very important for orthodontists to increase communication and find ways to let patients understand that the dental office is safe, and that many precautions are taken to avoid spreading COVID-19, because, as in other medical fields, some dental problems cannot be left untreated and require immediate treatment.

## Figures and Tables

**Table 1:** Question correlations.

During the pandemic did you go to your orthodontist in his office more or less than before	
Question	Significance
Did you start your orthodontic treatment during the pandemic?	*p* < 0.01
Did the pandemic influence your decision to go to the dental office to see an orthodontist?	*p* < 0.01
Did you feel a change in the orthodontist availability in satisfying your requests during the pandemic?	*p* < 0.01
Did the pandemic influence the importance that you usually attribute to your smile appearance?	
Did the pandemic influence the care that you usually give to your teeth health?	*p* < 0.01
Did the obligatory use of the facial mask to avoid COVID-19 infection influence your decision to have an orthodontic treatment?	*p* < 0.01
Did you start your orthodontic treatment during the pandemic?	
Did the pandemic influence the care that you usually give to your teeth health?	*p* < 0.05
Did the uncertain economic situation caused by the pandemic, influence your decision to have an orthodontic treatment?	N.S
Did you decide to postpone or not to have an orthodontic treatment because of the current economic situation?	N.S
Did the uncertain economic situation caused by the pandemic influence your decision to have an orthodontic treatment?	
Did the pandemic push you to ask for a specific kind of orthodontic device	*p* < 0.01

**Table 2:** Questionnaire answers. N° of visits is expressed as mean ± SD, discrete answers are expressed as absolute (percentage).

How many times did you go to see your orthodontist in his office during the last year (the pandemic)?	5.23 + 4.3
During the pandemic did you go to your orthodontist in his office more or less than before?	
Less	46 (29.3%)
The same	72 (45.9%)
More	39 (24.8%)
Did you start your orthodontic treatment during the pandemic?	
No	67 (42.1%)
Yes	92 (57.9%)
Did the pandemic influence your decision to go to the dental office to see an orthodontist?	
It discouraged you	18 (11.4%)
It did not influence your Decision	121 (76.6%)
It encouraged you	19 (12%)
Did the obligatory use of the facial mask to avoid COVID-19 infection influence your decision to have an orthodontic treatment?	
No	129 (81.1%)
Yes	30 (19.9%)
Did the pandemic push you to ask for a specific kind of orthodontic device?	
No	147 (92.5%)
Yes	11 (6.9%)
Did the pandemic influence the importance that you usually attribute to your smile appearance?	
The importance decreased	7 (4.4%)
Unchanged	132 (83%)
The importance increased	20 (12.6%)
Did the pandemic influence the care that you usually give to your teeth health?	
It decreased	9 (5.7%)
Unchanged	120 (75.5%)
It increased	30 (18.9%)
Did your oral hygiene change during the pandemic?	
It got worse	10 (6.3%)
Unchanged	103 (65.6%)
It improved	44 (28.0%)
Did the uncertain economic situation caused by the pandemic, influence your decision to have an orthodontic treatment?	
No	134 (84.3%)
Yes	24 (15.1%)
Did you decide to postpone or not to have an orthodontic treatment because of the current economic situation?	
NO	143 (89.9%)
Yes	16 (10.1%)
Did you feel a change in the orthodontist availability in satisfying your requests during the pandemic?	
The availability decreased	36 (22.9%)
The availability did not change	113 (72%)
The availability increased	8 (5.1%)

## Data Availability

The data that support the findings of this study are available from the corresponding author (Sabina Saccomanno) upon reasonable request.
